# Supplementary material for: FIP200 restricts RNA virus infection by facilitating RIG-I activation
Source: Commun Biol. 2021 Jul 29;4:921. doi: 10.1038/s42003-021-02450-1 (PMC8322336; doi:10.1038/s42003-021-02450-1)
Supplement: Supplementary file 2 — Supplementary Information [file 42003_2021_2450_MOESM2_ESM.pdf]

Supplemental Figure Legends

Suppl. Fig. 1. FIP200 co-localizes and interacts with RIG-I. (**a**) The outline of AP-MS. (**b**) FIP200 and the high confidence candidate interacting proteins (HCIP) are shown as square and circles, respectively. The blue line indicates a previously known interaction, and the red line indicates a new interaction. The arrow indicates RIG-I. (**c**) FIP200-FLAG was co-transfected with RIG-I-HA into HEK293 cells. After 48 h, cell lysates were immunoprecipitated with an anti-FLAG antibody and blotted as indicated. (**d**) FIP200-FLAG and RIG-I-HA were transfected into A549 cells. After 48 h, cells were fixed and stained as indicated. FLAG: red; HA: green; DAPI, blue. Bar = 10 μM. (**e**) A549 cells were transfected with 1 μg ml^{-1} poly(I:C). Two hours later, cells were treated Mitotracker Red for 15 min followed by fixation. The proximity ligation assays were performed. Green: PLA signal; red: mitochondrial tracker; blue: a nuclear stain. Arrows indicate PLA signals in the mitochondria. Bar = 10 μM. (**f**) FIP200-HA was co-transfected with FLAG-tagged MDA5 and the indicated mutants into HEK293 cells. After 48 h, cell lysates were immunoprecipitated with an anti-FLAG antibody and blotted as indicated. (**g**) The Myc-tagged 2CARD (2CARD-Myc) was transfected with vector, FIP200-FLAG, or FLAG-tagged ATG domain into HEK293 cells. After 48 h, cell lysates were immunoprecipitated with an anti-FLAG antibody and blotted as indicated. The arrowhead indicates FLAG-tagged ATG. The arrow indicates a possible SDS-resistant dimer of ATG. (**h**) Purified recombinant His-tagged ATG domain of FIP200 was mixed with GST, 2CARD-GST *in vitro* at 4 °C for 16 h. Then, His pulldown assay was performed and blotted as indicated.

Suppl. Fig. 2. FIP200 is required for RIG-I signaling in fibroblasts. (**a**) The cell lysates of *Fip200*$^{f/f}$ and *Fip200*$^{-/-}$ MEFs were blotted as indicated. (**b**) MTT assays of *Fip200*$^{f/f}$ and *Fip200*$^{-/-}$ MEFs. All experiments were biologically repeated three times. Data represent means ± s.d. of three independent experiments. (**c**) *Fip200*$^{f/f}$ and *Fip200*$^{-/-}$ MEFs were transfected with the

indicated amount of poly(I:C). After 16 h, The supernatants were collected for IFNα ELISA assays. All experiments were biologically repeated three times. Data represent means ± s.d. of three independent experiments. The *P* value was calculated (two-tailed Student's *t*-test) by comparison with the *Fip200*$^{f/f}$ cells. **P* < 0.05, ****P* < 0.001. (**d**) *Fip200*$^{f/f}$ and *Fip200*$^{-/-}$ MEFs were transfected with 1 μg ml^{-1} of poly(I:C) for the designated times. The supernatants were collected for IFNβ ELISA assays. All experiments were biologically repeated three times. Data represent means ± s.d. of three independent experiments. The *P* value was calculated (two-tailed Student's *t*-test) by comparison with the *Fip200*$^{f/f}$ cells. **P* < 0.05. (**e-f**) *Fip200*$^{f/f}$ and *Fip200*$^{-/-}$ MEFs were stimulated with 1 μg ml^{-1} poly(I:C) for indicated times. Real-time PCR was performed to determine the relative mRNA levels of IFNβ (**e**) and IRF7 (**f**). All experiments were biologically repeated three times. Data represent means ± s.d. of three independent experiments. The *P* value was calculated (two-tailed Student's *t*-test) by comparison with the *Fip200*$^{f/f}$ cells. **P* < 0.05, ***P* < 0.01. (**g**) MTT assays of HEK293 and two indicated FIP200 knockout cell lines. All experiments were biologically repeated three times. Data represent means ± s.d. of three independent experiments. (**h**) The cell lysates of HEK293 and two FIP200 knockout cell lines were blotted as indicated. (**i-j**) Wild type and FIP200 knockout HEK293 cells were stimulated with 1 μg ml^{-1} poly(I:C) for indicated times. Real-time PCR was performed to determine the relative mRNA levels of OASL (**i**) and IFIT1 (**j**). All experiments were biologically repeated three times. Data represent means ± s.d. of three independent experiments. The *P* value was calculated (two-tailed Student's *t*-test) by comparison with wild type cells. **P* < 0.05.

Suppl. Fig. 3. FIP200 is essential for RIG-I signaling in macrophages. (**a**) The cell lysates of wild type and FIP200 knockout RAW 264.7 macrophages were blotted as indicated. (**b**) MTT assays of wild type and FIP200 knockout RAW 264.7 macrophages. All experiments were biologically repeated three times. Data represent means ± s.d. of three independent

experiments. (**c-d**) Wild type and FIP200 knockout RAW264.7 macrophages were stimulated with the indicated amount of high molecular weight poly(I:C) (c) or poly(A:U) (**d**). After 16 h, the supernatants were collected for IFNβ ELISA assays. All experiments were biologically repeated three times. Data represent means ± s.d. of three independent experiments. The P value was calculated (two-tailed Student's t-test) by comparison with wild type cells. *$P < 0.05$, **$P < 0.01$. (**e**) Wild type and FIP200 knockout RAW 264.7 macrophages were infected with 1 MOI of IAV delNS1. After 16 h, the supernatants were collected for IFNβ ELISA assays. All experiments were biologically repeated three times. Data represent means ± s.d. of three independent experiments. The P value was calculated (two-tailed Student's *t*-test) by comparison with wild type cells. **$P < 0.01$.

Suppl. Fig. 4. The autophagy function is dispensable for FIP200-mediated RIG-I activation. (**a-b**) Wild type HEK293 cells, FIP200 knockout cells reconstituted with full-length FIP200, delATG, or delClaw were stimulated with 1 μg ml^{-1} poly(I:C) for designated times. Real-time PCR was performed to determine the relative mRNA levels of IP10 (**a**) and RANTES (**b**). All experiments were biologically repeated three times. Data represent means ± s.d. of three independent experiments. The P value was calculated (two-tailed Student's *t*-test) by comparison with HEK293 cells. *$P < 0.05$, **$P < 0.01$. (**c**) Two hundred ng of FLAG-tagged FIP200, FIP200-4A, or ULK1 was transfected with 20 ng of FLAG-tagged RIG-I and 20 ng of pRL-SV40, together with 200 ng of pISRE-Luc or NF-κB-Luc into HEK293 cells. After 48 h, cells were collected and the ratio of firefly luciferase to *Renilla* luciferase was calculated to determine the relative activity of IFN reporter. All experiments were biologically repeated three times. Data represent means ± s.d. of three independent experiments. The P value was calculated (two-tailed Student's *t*-test) by comparison with the RIG-I/vector transfection. *$P < 0.05$, **$P < 0.01$.

Suppl. Fig. 5. FIP200 limits RNA virus infection. (**a**) FIP200 wild type and knockout HEK293 cells were infected with VSV-Luc for 16 h. Luciferase activity was measured to determine relative viral infection activity. All experiments were biologically repeated three times. Data represent means ± s.d. of three independent experiments. The *P* value was calculated (two-tailed Student's *t*-test) by comparison with wild type cells. **P* < 0.05, ***P* < 0.01. (**b**) *Fip200*$^{f/f}$ and *Fip200*$^{-/-}$ MEFs were infected with the VSV carrying a GFP gene (VSV-GFP) for 16 h. The relative infection was determined by the ratio of GFP positive cells and summarized in the right panel. Bar = 50 μM. Data represent means ± s.d. of three independent experiments. The *P* value was calculated (two-tailed Student's t-test) by comparison with the wild type cells. **P* < 0.05. (**c**) Wild type and FIP200 knockout RAW264.7 macrophages were infected with designated amounts of VSV. After 16 h, cell lysates were collected and blotted as indicated. (**d**) ATG5 wild type and knockout MEFs were infected with VSV-GFP for 16 h. The relative infection was determined by the ratio of GFP positive cells and summarized in the right panel. Bar = 50 μM. Data represent means ± s.d. of three independent experiments. The *P* value was calculated (two-tailed Student's t-test) by comparison with the wild type cells. **P* <0.05. (**e**) Wild type HEK293 cells, FIP200 knockout cells, and FIP200 knockout cells reconstituted with full-length FIP200 or the 4A mutant were infected with 0.1 MOI of VSV-Luc for 16 h. Luciferase activity was measured to determine relative viral infection activity. Data represent means ± s.d. of three independent experiments. The *P* value was calculated (two-tailed Student's *t*-test) by comparison with the wild type cells. ***P* < 0.01. (**f**) Wild type and FIP200 knockout RAW264.7 macrophages were infected with VACV-Luc for 16 h. Luciferase activity was measured to determine relative viral infection activity. Data represent means ± s.d. of three independent experiments.

Suppl. Fig. 6. FIP200 is required for RIG-I signaling and host defense *in vivo*. (**a-b**) The

BMDMs of *Fip200*$^{f/f}$ and *Fip200*$^{f/f}$;LysM-Cre mice were stimulated with 1 μg ml^{-1} of poly(I:C) for indicated times. Real-time PCR was performed to determine the relative mRNA levels of IP10 (**a**) and RANTES (**b**). Data represent means ± s.d. of three independent experiments. The *P* value was calculated (two-tailed Student's *t*-test) by comparison with the wild type cells. $^*P < 0.05$, $^{**}P < 0.01$. (**c**) Histomicrographs comparing pulmonary lesions between *Fip200*$^{f/f}$ and *Fip200*$^{f/f}$;LysM-Cre mice 7 d after VSV infection. *Fip200*$^{f/f}$;LysM-Cre mouse exhibited a marked diffuse interstitial pneumonia whereas lung tissue obtained from *Fip200*$^{f/f}$ mouse was essentially normal. Arrows point to regions of vasculitis with lymphocyte infiltrate. Bar = 100 μM.

Suppl. Fig. 7. FIP200 regulates the release and oligomerization of 2CARD. (**a**) Purified recombinant Hel-CTD-FLAG was mixed with GST, 2CARD-GST or 2CARD-GST plus ATG-His at 4 °C. After 16 h, GST pull-down assay was performed. Arrow indicates the 2CARD-GST. Arrowhead indicates the cleaved GST from 2CARD-GST during extraction and purification from *E. coli*. (**b**) HA-tagged ubiquitin (Ub-HA), GFP-tagged FIP200 (FIP200-GFP), and RIG-I-FLAG were transfected into HEK293 cells in the indicated combinations. After 48 h, cell lysates were immunoprecipitated and blotted as indicated. (**c**) FIP200 wild type and knockout HEK293 cells were transfected with RIG-I-FLAG and Ub-HA. After 48 h, cell lysates were immunoprecipitated and blotted as indicated. (**d**) *Fip200*$^{f/f}$ and *Fip200*$^{-/-}$ MEFs were stimulated with 1 μg ml^{-1} poly(I:C) for 4 h. Then the cell lysates were separated by 15–55% sucrose density centrifugation. Fractions were blotted using the anti-RIG-I antibody. The fraction of thyroglobulin (660 kDa), a protein standard, was indicated. (**e**) HA-tagged ATG (ATG-HA) was co-transfected with vector or FLAG-tagged ATG (ATG-FLAG) into HEK293 cells. After 48 h, cell lysates were immunoprecipitated and blotted as indicated. (**f**) HA-tagged CC (CC-HA) was co-transfected with vector or FLAG-tagged CC (CC-FLAG) into HEK293 cells. After 48 h, cell lysates were immunoprecipitated and blotted as indicated. (**g**) FIP200-HA was co-transfected with vector,

FIP200-FLAG, or the indicated FIP200 mutants into HEK293 cells. After 48 h, cell lysates were immunoprecipitated and blotted as indicated. (**h-i**) Wild type HEK293 cells, FIP200 knockout cells, FIP200 knockout cells reconstituted with full-length FIP200 or the delCC mutant were stimulated with 1 μg ml^{-1} poly(I:C) for designated times. Real-time PCR was performed to determine the relative mRNA levels of IP10 (**h**) and RANTES (**i**). Data represent means ± s.d. of three independent experiments. The *P* value was calculated (two-tailed Student's *t*-test) by comparison with wild type HEK293 cells. $^*P < 0.05$, $^{**}P < 0.01$, $^{***}P < 0.001$. Right panel in (**h**) shows the expression of FIP200 and the indicated mutant in the reconstituted cells.

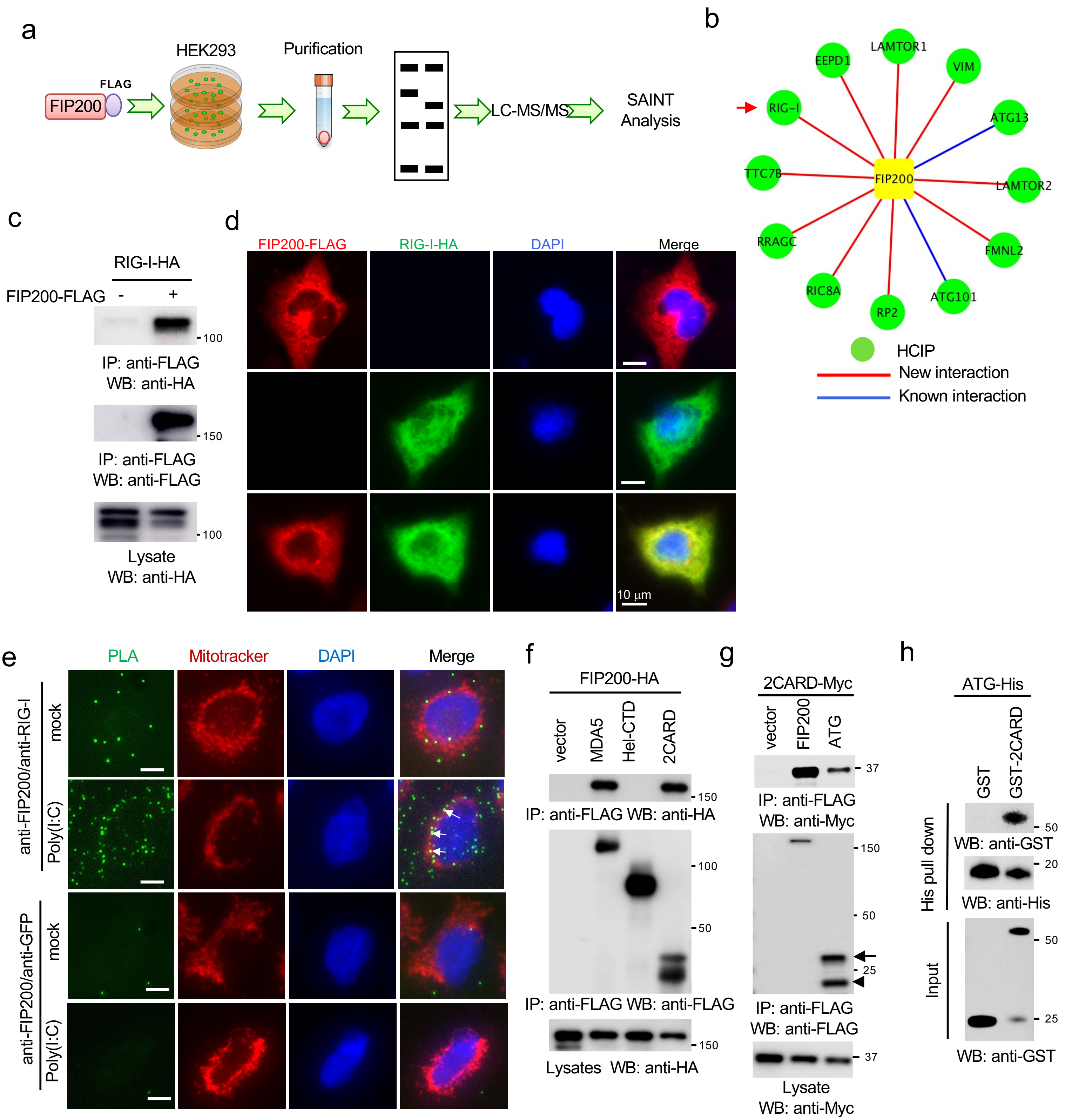

Suppl. Figure 1

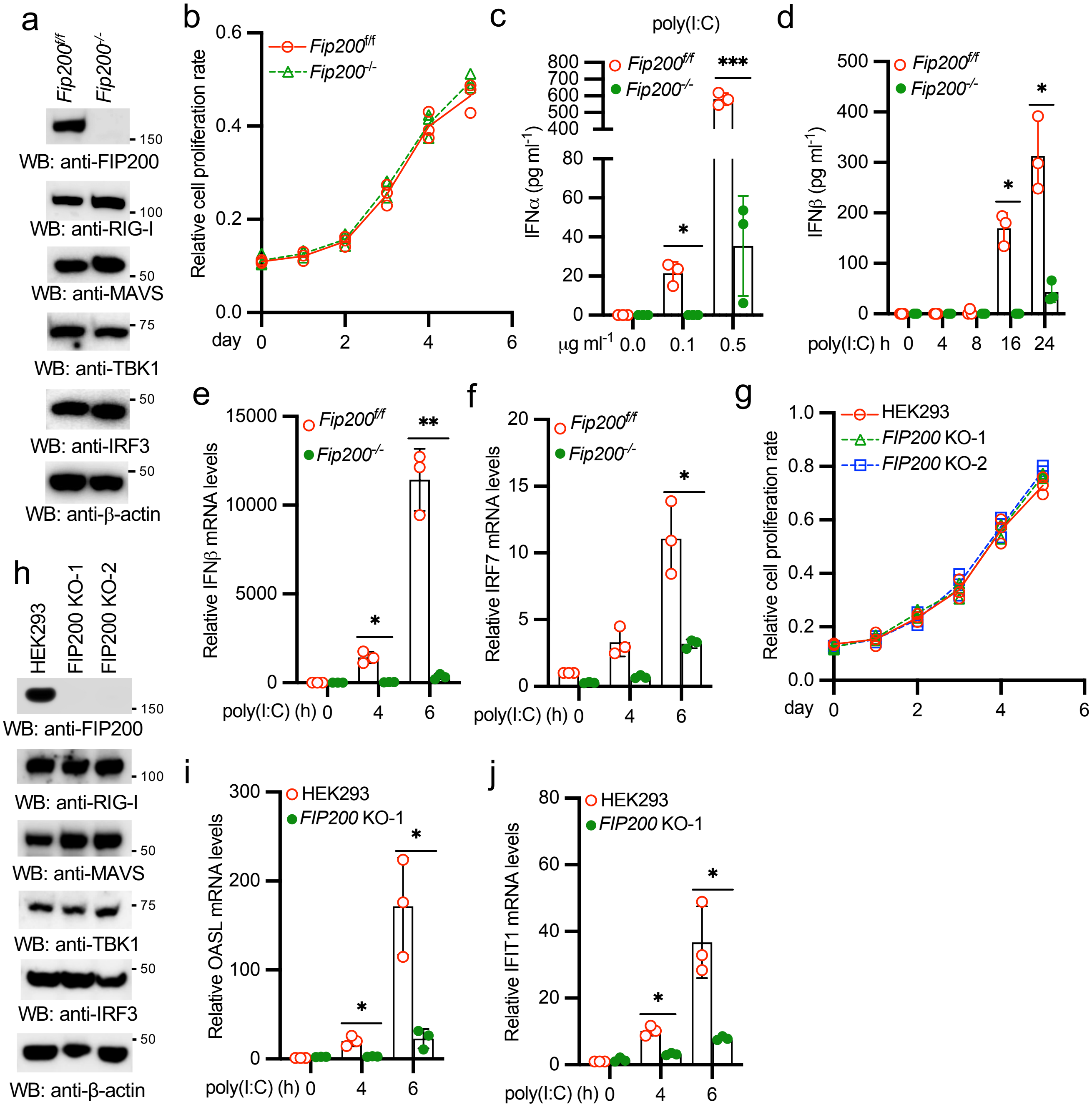

Suppl. Figure 2

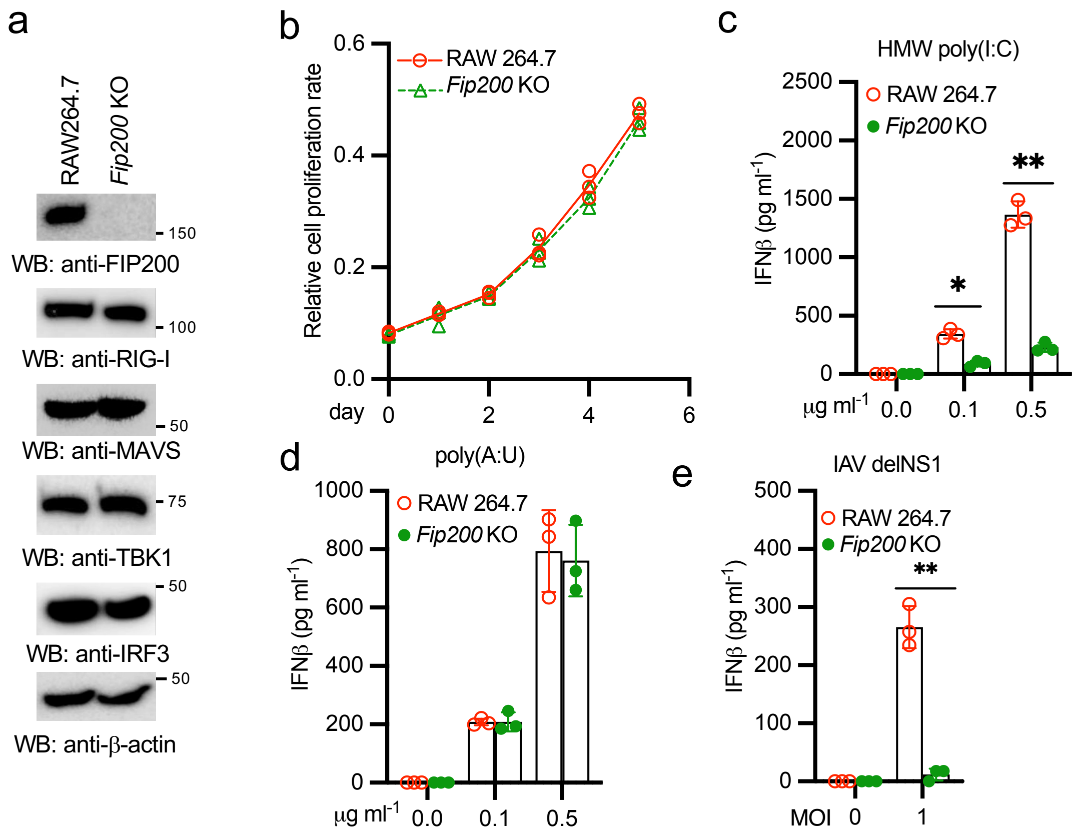

Suppl. Figure 3

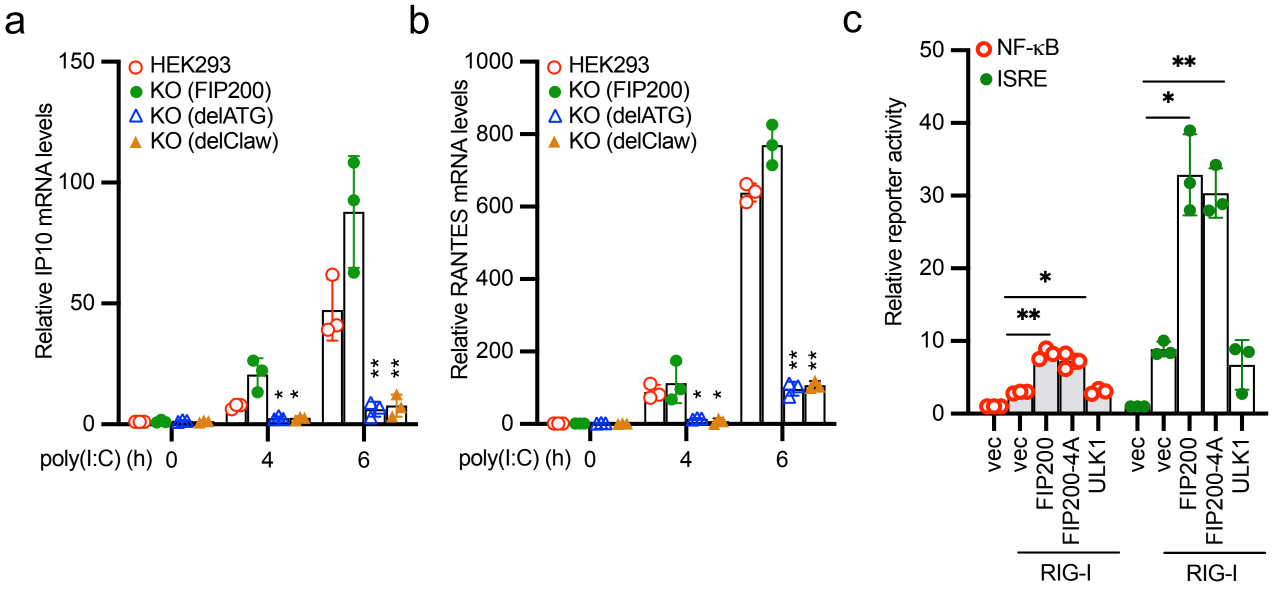

Suppl. Figure 4

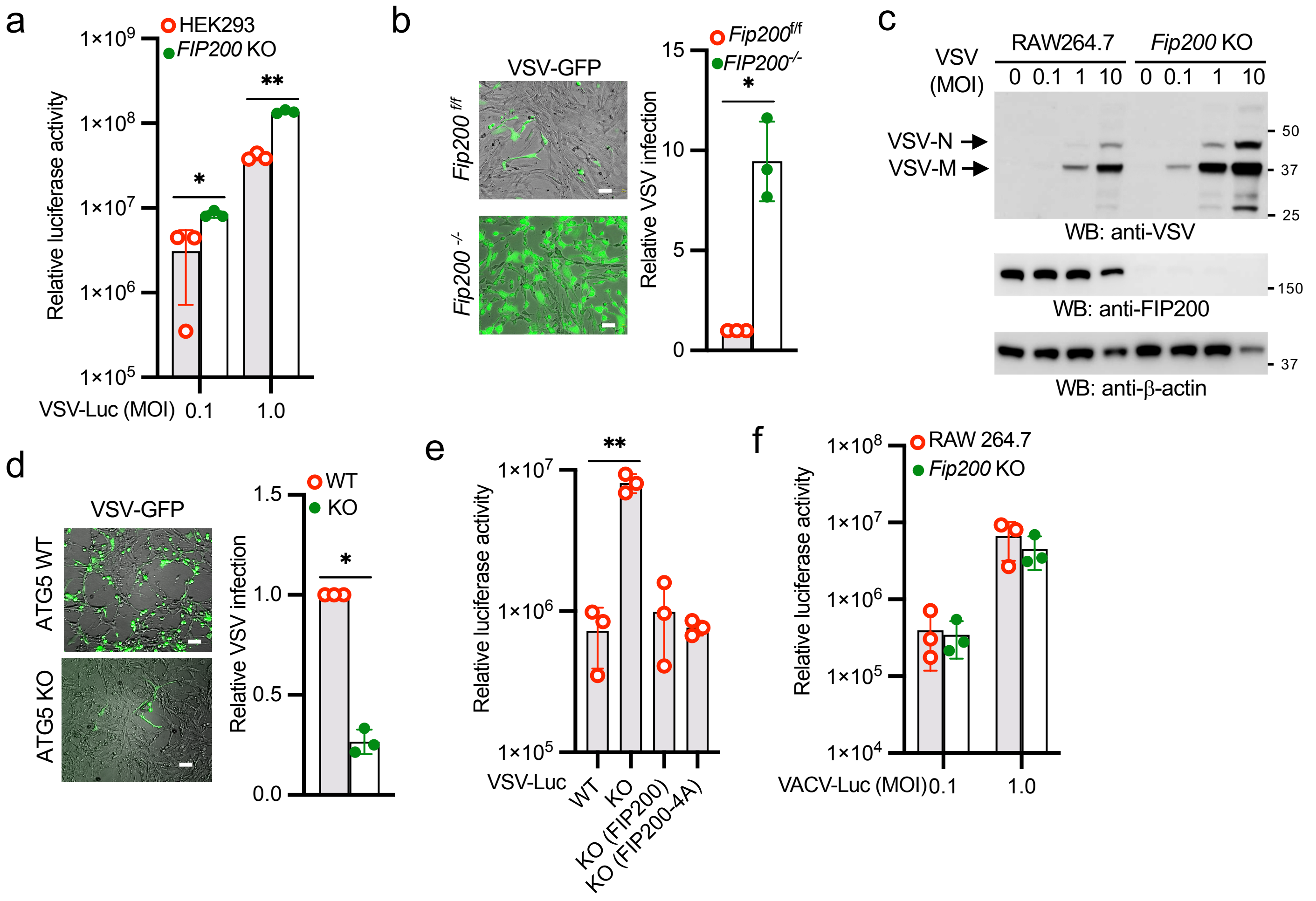

Suppl. Figure 5

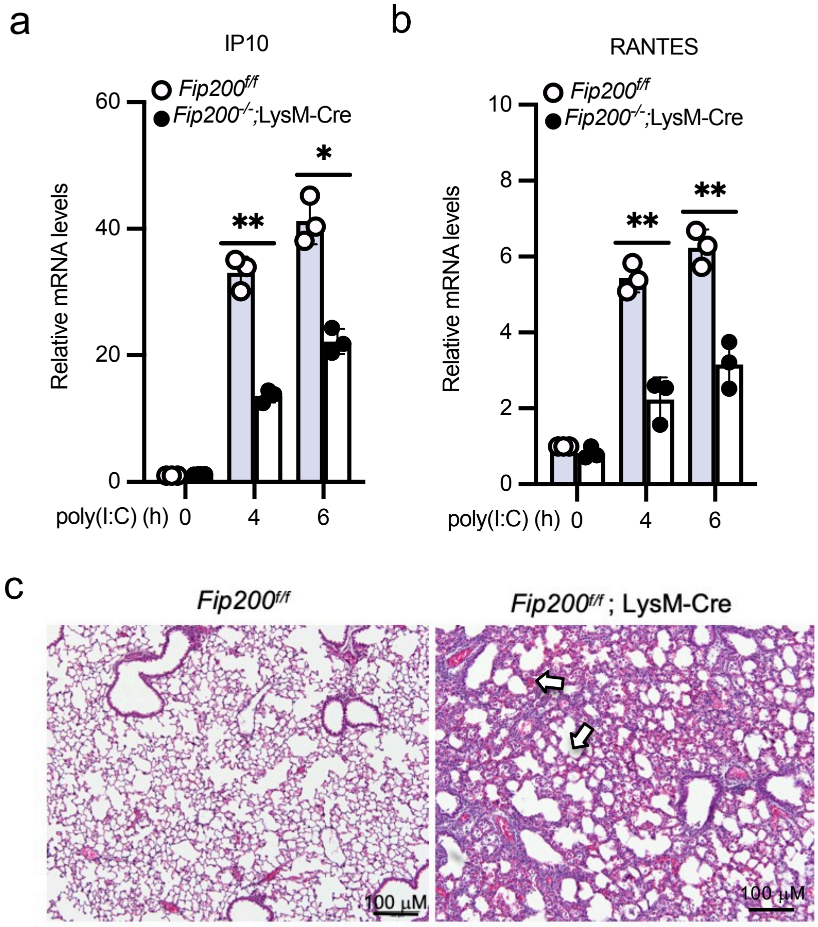

Suppl. Figure 6

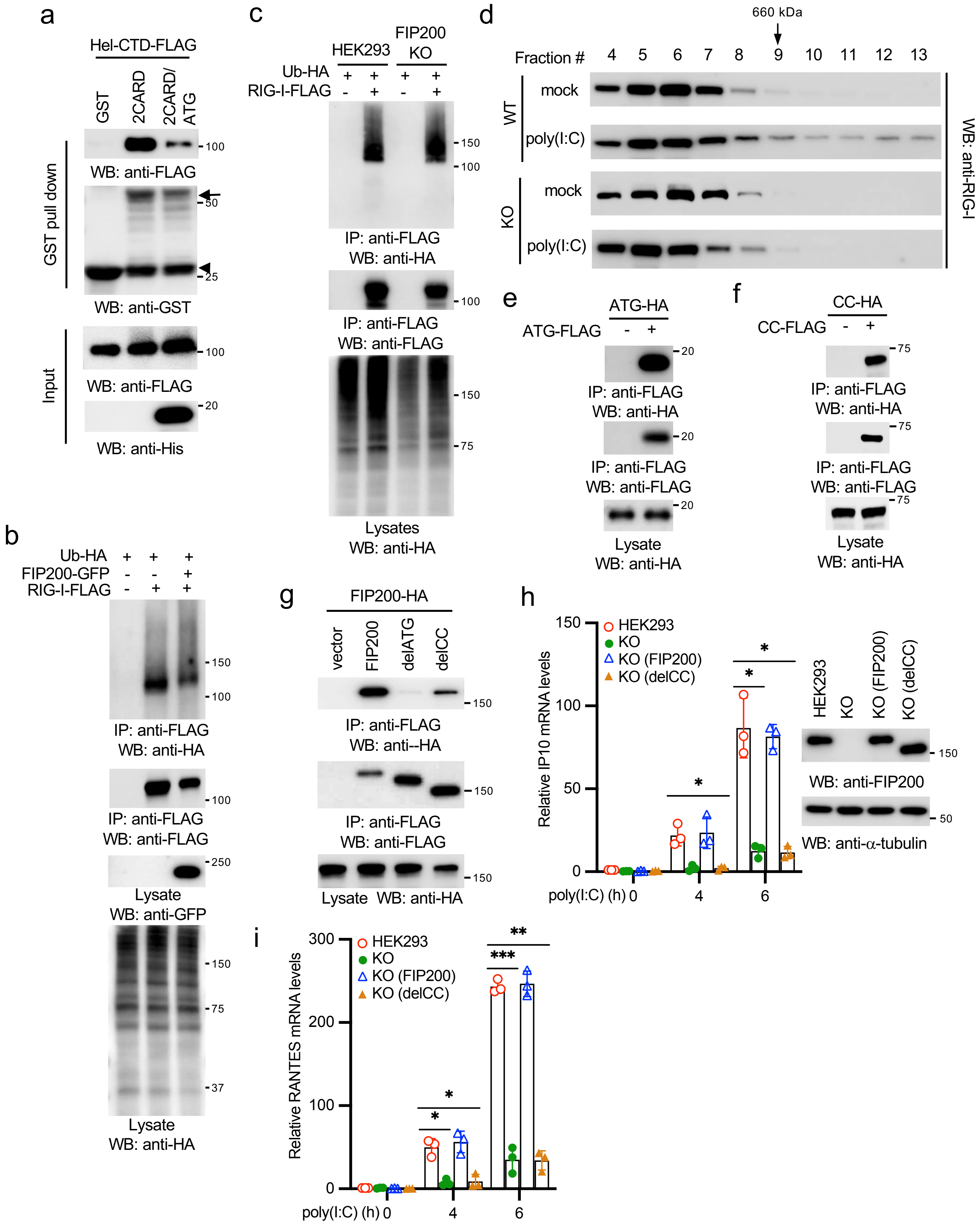

Suppl. Figure 7

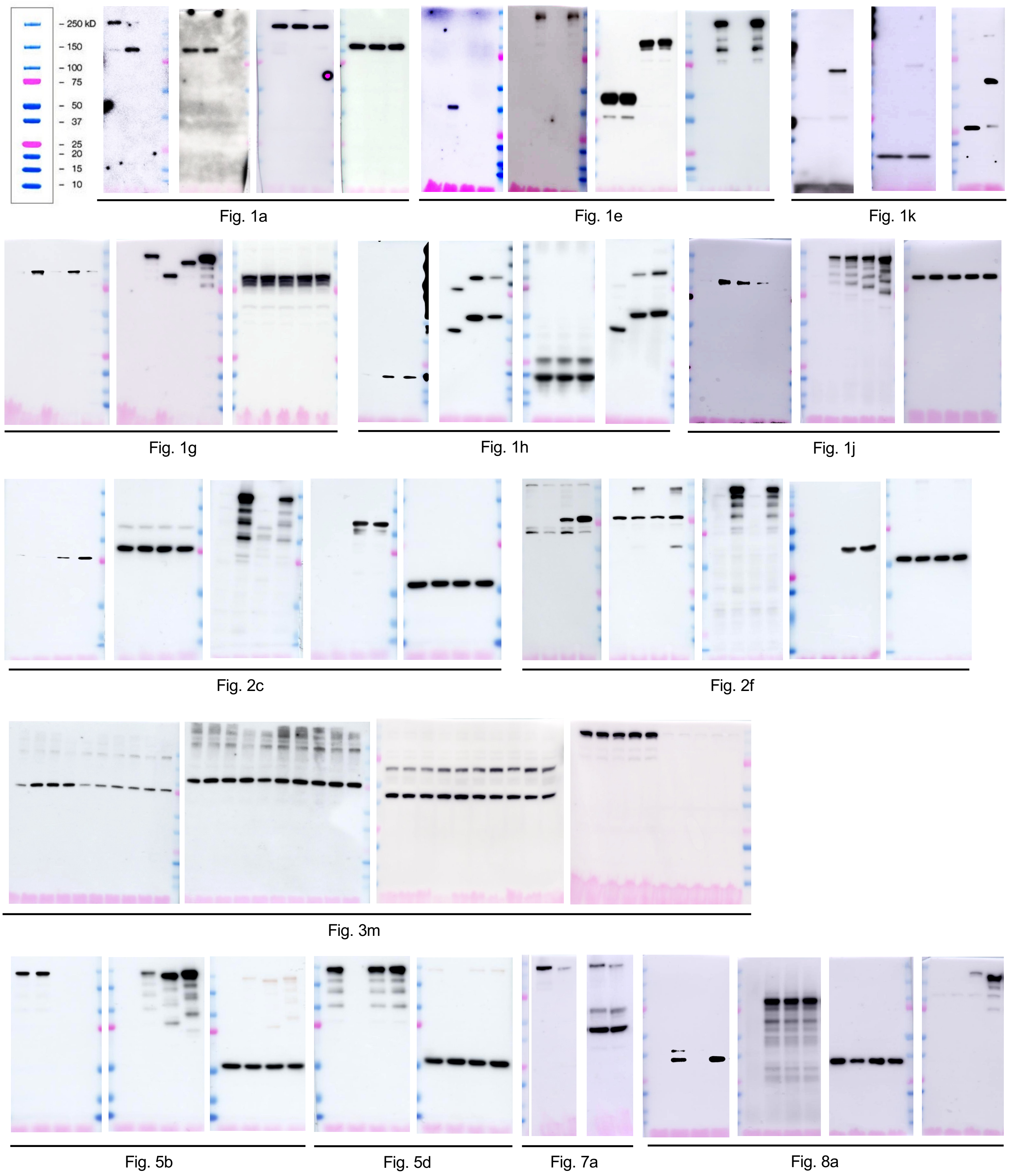

Suppl. Figure 8

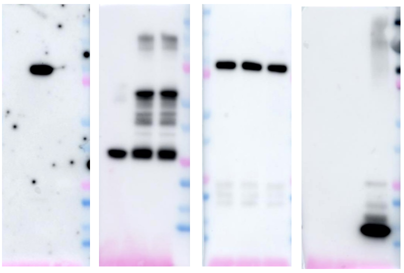

Fig. 8b

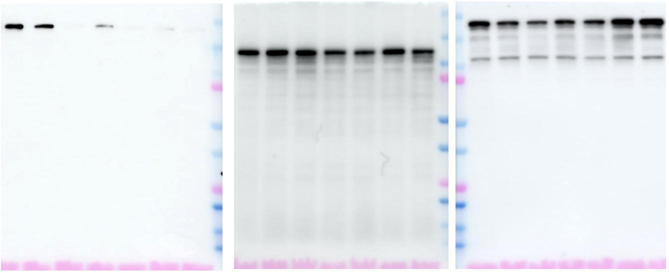

Fig. 8c

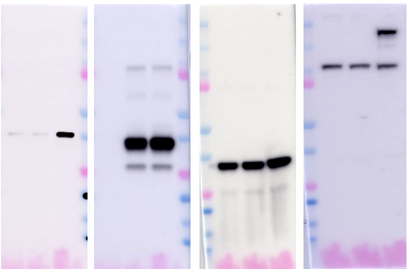

Fig. 8d

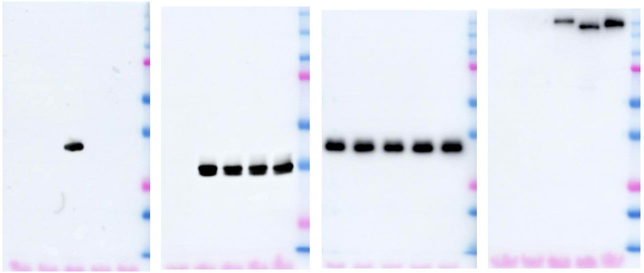

Fig. 8e

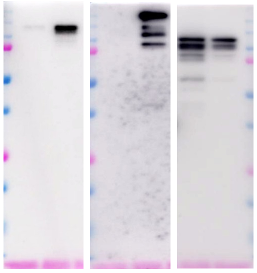

Suppl. Fig. 1c

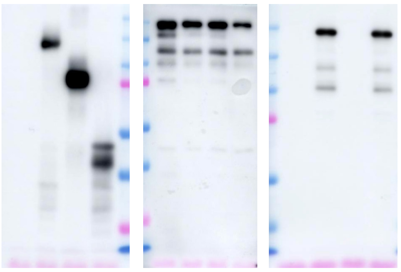

Suppl. Fig. 1f

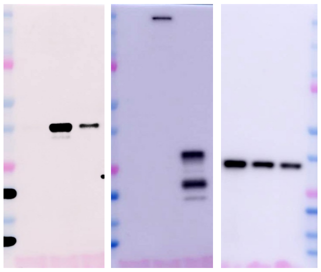

Suppl. Fig. 1g

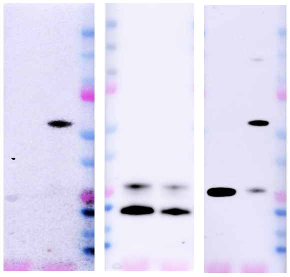

Suppl. Fig. 1h

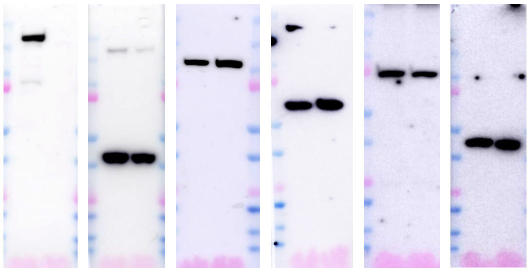

Suppl. Fig. 2a

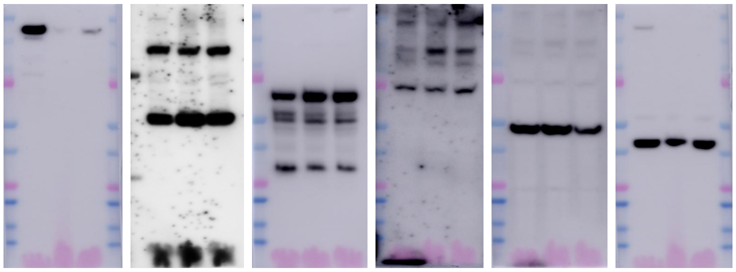

Suppl. Fig. 2h

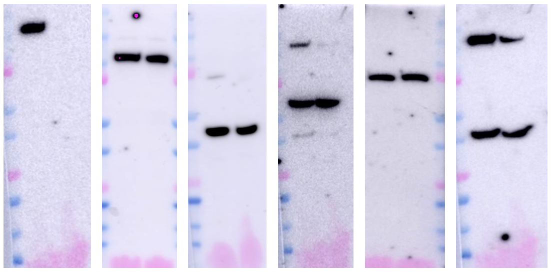

Suppl. Fig. 3a

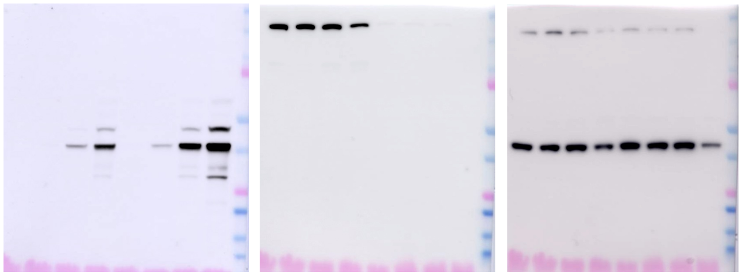

Suppl. Fig. 5c

Suppl. Figure 9

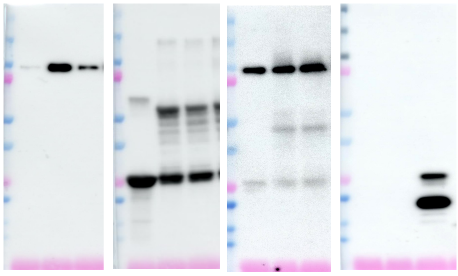

Suppl. Fig. 7a

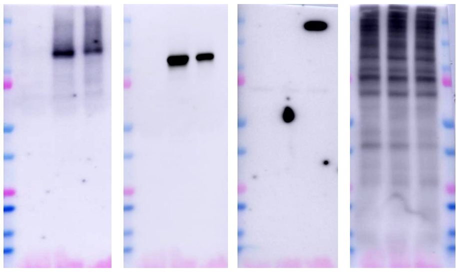

Suppl. Fig. 7b

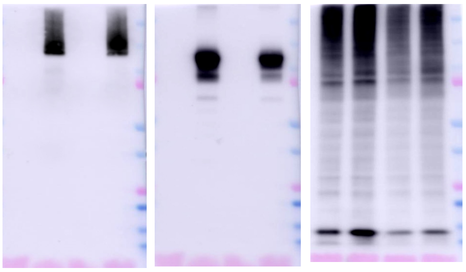

Suppl. Fig. 7c

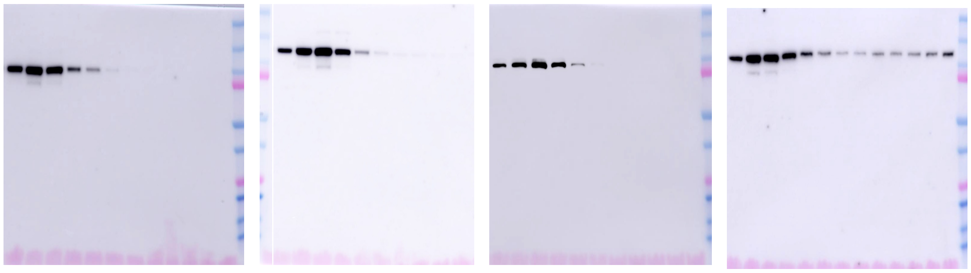

Suppl. Fig. 7d

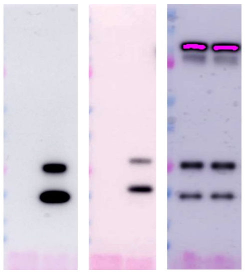

Suppl. Fig. 7e

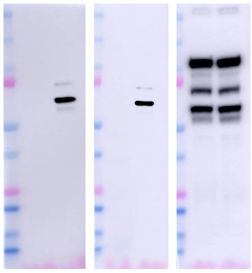

Suppl. Fig. 7f

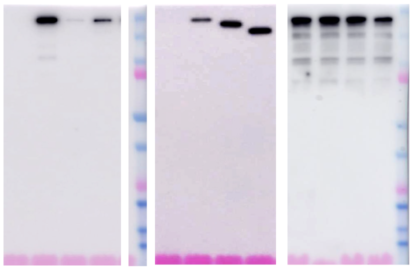

Suppl. Fig. 7g

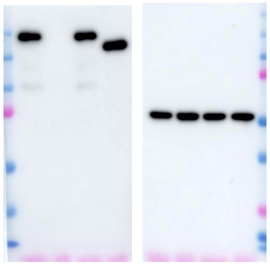

Suppl. Fig. 7h

Suppl. Figure 10
