# Supplementary material for: FIP200 restricts RNA virus infection by facilitating RIG-I activation
Source: Commun Biol. 2021 Jul 29;4:921. doi: 10.1038/s42003-021-02450-1 (PMC8322336; doi:10.1038/s42003-021-02450-1)
Supplement: Supplementary file 3 — Description of Supplementary Files [file 42003_2021_2450_MOESM3_ESM.pdf]

Description of Additional Supplementary Files

File name: Supplementary Data 1

Description: The complete mass spectrometry data of FIP200 protein complex.

File name: Supplementary Data 2

Description: All source data for Figures.
